# Development and Piloting of an Algorithm to Select Older Patients for Different Types of Medication Review

**DOI:** 10.3389/fphar.2019.00217

**Published:** 2019-03-19

**Authors:** Stijn Crutzen, Jan Schuling, Jacqueline G. Hugtenburg, Monique Verduijn, Martina Teichert, Katja Taxis, Petra Denig

**Affiliations:** ^1^Department of Clinical Pharmacy and Pharmacology, University Medical Center Groningen, University of Groningen, Groningen, Netherlands; ^2^Department of General Practice, University Medical Center Groningen, University of Groningen, Groningen, Netherlands; ^3^Department of Clinical Pharmacology and Pharmacy, VU University Medical Center, Amsterdam, Netherlands; ^4^Department of Guideline Development and Research, The Dutch College of General Practitioners, Utrecht, Netherlands; ^5^Department of Clinical Pharmacy and Toxicology, Leiden University Medical Center, Leiden, Netherlands; ^6^Unit of PharmacoTherapy, Epidemiology and Economics, Groningen Research Institute of Pharmacy, University of Groningen, Groningen, Netherlands

**Keywords:** medication reviews, polypharmacy, patient selection, aged, primary care

## Abstract

**Aim:** To develop and pilot an algorithm to select older people for different types of medication review based on their case complexity.

**Methods:** Experts rated complexity of patient cases through a Delphi-consensus method. The case characteristics were included in a regression model predicting complexity to develop a criteria-based algorithm. The algorithm was piloted in four community pharmacies with 38 patients of high and low complexity. Pharmacists conducted medication reviews according to their personal judgment and rated the patients' complexity. Time needed for reviewing and number of interventions (proposed and implemented) were assessed. Feasibility was evaluated with in-depth interviews.

**Results:** We developed the algorithm with 75 cases proceeding from patients in average 79 years old and using 10 prescribed medications. The regression model (adjusted *R*^2^ = 0.726, *P* < 0.0001) resulted in the following criteria for the algorithm: “number of medications” × 1 + “number of prescribers” × 3 + “recent fall incident” × 7 + “does not collect own medication” × 4. The pharmacists performed advanced medication reviews with all patients. The time needed to perform the medication review did not differ significantly according to case complexity (76.9 min for high complexity; 66.1 min for low complexity). Agreement between the algorithm scores and the pharmacists' ratings on complexity degree was slight to moderate (Kappa 0.16–0.42). The pharmacists had mixed opinions about the feasibility of applying the algorithm, particularly regarding the criterion “fall incidents.”

**Conclusion:** We developed an algorithm with four criteria that distinguished between high and low complexity patients as rated by experts. Additional validation steps are needed before implementation.

## Introduction

Aging and polypharmacy are associated with an increased risk of medication-related harm (Leendertse et al., 2008; Kaufmann et al., 2015). Medication reviews have shown to reduce the number of inappropriately used medications and resultant drug-related problems (Holland et al., 2008). Medication reviews have been implemented in many countries (Bulajeva et al., 2014), although effects on outcomes like hospital admissions or quality of life have not been consistently demonstrated (Holland et al., 2005, 2008; Lenaghan et al., 2007; Hatah et al., 2014; Huiskes et al., 2017; Hurmuz et al., 2018). Three types of medication reviews have been put forward (Blenkinsopp et al., 2012; Griese-Mammen et al., 2018). These are simple reviews based on the pharmacy medication history only, intermediate reviews for which the patient provides additional information and advanced reviews that make use of more extensive clinical information. Currently there are no uniform or evidence-based strategies to select patients for different types of medication reviews. Commonly used criteria to select patients for medication reviews include an age of 65 years or older and the chronic use of multiple medications (Huiskes et al., 2017). In the Netherlands, advanced clinical medication reviews are recommended for all older patients with a high case complexity (Nederlands Huisartsen Genootschap, 2012). Case complexity refers to the patient's overall status, including polypharmacy, multimorbidity, and psycho-social problems (Huyse et al., 2007). The Dutch guideline states that patients should be selected for an advanced medication review if they are ≥65 years of age, take ≥5 chronic medications and have at least one additional risk factor. These risk factors include having a reduced kidney function, a diminished cognition, an increased fall risk, low medication adherence, an unplanned hospitalization in the past year, or living in a nursing home.

Selection of patients according to the Dutch guideline recommendations turned out to be not feasible in practice. Information regarding the additional risk factors is not routinely available to the community pharmacists. Without this information large numbers of patients were selected, though not all needed an advanced medication review (Pharmaceutisch Weekblad, 2015). The laborious nature of advanced reviews unnecessarily burdens healthcare professionals. Therefore, the Dutch Healthcare Inspectorate decided that advanced medication reviews should at least be conducted for all patients of ≥75 years of age, with ≥7 chronic medications and a decreased kidney function (Landelijke Huisartsen Vereniging, 2016; Pharmaceutisch Weekblad, 2016). This pragmatic approach received criticism because it would lead to the exclusion of a large group of older patients for whom a medication review could be beneficial. A new approach with different criteria is needed that allows patients to be allocated to different types of medication reviews.

The aim of our study is firstly to develop a criteria-based algorithm for the allocation of older people (≥65 years and with ≥5 medications) for simple or advanced medication reviews, based on their case complexity. Secondly, we piloted the algorithm in the community pharmacy setting.

## Methods

### Algorithm Development

We used judgment analysis to derive the criteria predicting case complexity to be included in the algorithm (Cooksey, 1996). Two panels of experts provided patient cases and rated their complexity. Panel A consisted of four community pharmacists and four general practitioners (GPs) and panel B consisted of five community pharmacists and three GPs.

#### Patient Cases

The experts were asked to submit four to six cases of patients using at least five chronic medications with varying complexity. High complexity was defined as a case for which existing treatment guidelines cannot be easily applied and an advanced medication review is required to optimize treatment. Low complexity was defined as a case for which existing treatment guidelines can be applied as part of a simple medication review in order to optimize treatment. The experts completed a structured case report form with case characteristics (Appendix I). This consisted of the patient's medication, morbidity, laboratory measurements, demographics, estimated medication use issues, estimated beliefs about medication and other possible risk factors for drug-related problems such as diminished cognition. The goal was to obtain 80 cases; using the rule of 10 subjects per predictor, this would be sufficient to develop an algorithm with eight criteria using regression modeling (Morgan et al., 2007). To achieve this number, we used all cases provided by the experts and generated additional cases. We modified a number of characteristics from a subset of randomly selected experts' cases. Level of education, eyesight, age, sex, and medication intake issues were systematically changed. Also, we exchanged one health issue and its corresponding medication with another health issue and its corresponding medication. All additional cases were checked by two researchers to ensure they remained realistic.

#### Expert Ratings

All cases were rated by the two expert panels using a modified Delphi method. For this, each panel rated the complexity of cases provided by the opposite panel on a 9-point Likert scale (1 = low complexity 9 = high complexity). Consensus per case was calculated according to the Interpercentile Range Adjusted for Symmetry method (Fitch et al., 2001). This method corrects for outliers by using the range between the 0.3 and the 0.7 percentile instead of the full range. A correction factor is used to adjust for symmetry of the ratings. Cases for which there was no consensus were discussed in a consensus meeting. A case was presented, the experts explained and discussed why they had given a specific rating of complexity, and after the discussion the experts were asked whether they wanted to reconsider their rating. For each case, the median of the individual complexity ratings of the experts was calculated.

#### Building the Algorithm

The algorithm was built using all cases for which consensus had been reached. The median complexity rating was used as the outcome in linear regression models, where potential criteria were included as predictors. A wide range of clinical, medication and patient characteristics were included in the models, some of which could be proxies for criteria that may be difficult to measure. In total 48 potential criteria in 25 categories were tested as predictors (Appendix II). Predictors were clustered when they were derived from the same information or represented a similar concept. A complete case linear regression procedure was followed, with only one predictor from the same cluster included so as to avoid collinearity. Predictors with a *P*-value of 0.25 or less in the univariate testing were included in the multivariate regression model. Per cluster the predictor with the lowest *P*-value in univariate testing was included in the first model. Predictors were excluded from the model until excluding another predictor would reduce the adjusted *R*^2^ by ≥0.05. When a predictor was removed from the model during the backward stepwise process, alternative predictor(s) from the same cluster were tested. The weights of the predictors of the final regression model were multiplied by $\frac{1}{Lowest\;weight}$ and rounded to the nearest whole number. The final algorithm consisted of the weighted predictors (criteria). With this the algorithm scores were calculated where a higher score indicated higher complexity.

### Pilot Study

The algorithm was piloted in four community pharmacies. The algorithm scores were compared with complexity ratings of the pharmacists as well as experts. The feasibility of applying the algorithm in practice was assessed with in-depth interviews with the participating pharmacists. The medication review process was evaluated.

#### Setting

The four participating community pharmacies, located in the northern part of the Netherlands, offer standard pharmaceutical care, including the monitoring of drug-drug interactions, contra-indications, along with dose control, and patient counseling. Furthermore, they have implemented advanced medication reviews in accordance with the Dutch guideline (van der Meer et al., 2015). All pharmacists routinely collaborate with the GPs including regular pharmacotherapy audit meetings.

#### Patient Selection and Medication Review

Patients for whom a medication review is reimbursed in the Netherlands were eligible for the pilot study. Thus, eligibility was defined as ≥65 years, ≥5 chronic prescriptions and not having received a medication review in the previous 12 months. The pharmacists invited a random sample of 100 patients per pharmacy to participate. Informed consent was collected from patients who accepted to participate. Consenting patients completed a questionnaire either by mail or by phone to collect the additional information needed for the algorithm, that is whether they collect their own medication, the number of prescribers and whether they experienced a fall recently (Appendix III). For each patient, the number of used medication was calculated including all prescriptions for medication at the lowest level ATC code, excluding non-therapeutic ophthalmologicals (S01K, S01J, S02DC), caries prophylactic agents (A01AA), over-the-counter vitamins (belonging to A11), blood substitutes and perfusion solutions (B05), emollients, protective, and other dermatologicals (D02, D11) (ATC/DDD Index, 2018). After applying the algorithm to all respondents we randomly selected five from the highest tertile and five from the lowest tertile of the algorithm scores for a medication review in each pharmacy. We used a random number generator in excel 2010 for this selection. We expected that patients from the highest tertile represented patients with a high complexity and patients from the lowest percentile represented patients with a low complexity. We selected five patients per complexity degree and per pharmacy to comply with time constraints. The pharmacists were instructed to perform an advanced medication review as usual for patients with a high algorithm score and were allowed to conduct any type of medication review for low complexity patients based on their clinical judgment.

#### Agreement

The participating community pharmacists were asked to rate case complexity of the selected patients on a 9-point Likert scale (1 = low complexity, 9 = high complexity). The need to perform a medication review for these patients was rated on a separate 9-point Likert scale. Similarly, for each case a pharmacist and a GP from the expert panels rated the complexity of the case and the need for a medication review. These ratings were averaged to provide one expert rating per case. Kappa statistics were used to assess the agreement on the complexity degree (high or low) between the algorithm scores and the community pharmacists' ratings. Similarly, this was done for the agreement between the algorithm scores and the experts' ratings. Different cut-off points were tested for the ratings on the 9-point scale, for which the agreement and Kappa were calculated. The Kappa values were interpreted as followed: <0.00 (poor), 0.00–0.20 (slight), 0.21–0.40 (fair), 0.41–0.60 (moderate), 0.61–0.80 (substantial), and 0.81–1.00 almost perfect agreement (Fau and Koch, 1977). Finally, we assessed agreement between the complexity degree (high or low) indicated by the algorithm and the need for a medication review indicated by the participating pharmacist and the experts.

#### Pharmacists' Views on the Feasibility

To assess feasibility, pharmacists were asked to share their positive and negative views about the use of the new criteria as compared to the current criteria (primary outcome). In-depth interviews were held with the four participating pharmacists by SC. These were face-to-face interviews using a topic list which was developed to assess their views about the criteria of the algorithm and applying them in practice (two domains with eight topics) and explore the setting and procedures regarding the conduct of medication reviews (two domains with six topics). An overview of the topics can be found in Appendix IV. The interviews were recorded, transcribed and thematically coded by SC. A content analysis was conducted to summarize information about the setting in relation to the conducting of medication reviews. A deductive approach was used to attain thematic codes related to the participants' positive and negative views about various criteria and the feasibility of applying such criteria in practice. The data coding related to the setting was checked by KT and thematic codes related to criteria and feasibility were checked by PD.

#### Medication Review Evaluation

As process evaluation, we assessed the number of proposed and implemented interventions as well as the time needed for each step in the review of patients with high and low complexity scores. The steps were preparation, anamnesis, analysis, consultation with GP, and feedback to patient (van der Meer et al., 2015). For each patient with a medication review a structured case report form was completed by a pharmacist in training together with the pharmacist containing the following data: laboratory outcomes and morbidity from GP medical records, background information obtained during anamnesis in the pharmacy, the proposed and implemented interventions as documented in the pharmacy medication record. Per review step the pharmacist recorded the time involved. *T*-tests were used to compare the time investment of the pharmacists and the number of proposed and implemented interventions between patients with high and low complexity scores.

## Results

The 59 cases provided by the experts combined with 21 additional cases developed from these cases created a total of 80 cases, which were rated by the experts on complexity. In the first Delphi round, consensus was reached on 59 of the 80 cases, including 18 of the additional cases. During the second round consensus was reached on another 16 cases, including three additional cases. Consensus was not obtained for five cases, which were excluded from further analysis. The patient characteristics of the cases included in the algorithm development are shown in Table 1.

**Table 1:** Patients characteristics of the 75 cases with expert panel consensus.

**Variable**	**Mean/%**	***N***	**Range**
Age (years)	79	–	49–94
Weight (kg)	78	–	50–110
Male	48%	36	–
Number of medications	10	–	5–26
At least one unplanned hospitalization in the last 12 months	24%	18	–
Number of prescribers	1.9	–	1–4
Does not collect his or her own medication at the pharmacy	64%	47	–
Receives sachet packed medication	49%	35	–
QOF standard morbidity score (Carey et al., 2013)	1.6	–	0–4
QOF extended morbidity score (excluding renal disease) (Carey et al., 2013)	1.6	–	0–4
Mean complexity rating by expert panel	5.0	–	2–9

*QOF, Quality and Outcomes Framework*.

### Algorithm Development

A total of 14 potential criteria with a *p*-value of ≥0.25 in the univariate testing were included in the first regression model predicting the experts' complexity rating (Appendix II). An additional 10 alternative potential criteria were tested during the stepwise procedure. In the final model, four criteria remained which significantly predicted the complexity rating (*P* < 0.0001) with an adjusted *R*^2^ of 0.73 (Table 2). The regression model results were used to construct the algorithm by multiplying the coefficients with $\frac{1}{0.232}$ and rounding this to the nearest whole number. This resulted in the algorithm with the following four criteria and weighting:

**Table 2:** Case characteristics predicting complexity (final regression model).

**Case characteristics**	**Coefficient**	***P*-value**
Number of different medications	0.232	<0.001
Number of prescribers	0.756	<0.001
At least one fall incident in the last 12 months (1 = yes, 0 = no)	1.65	<0.001
Does collect his/her own medication at the pharmacy (0 = yes, 1 = no)	0.884	0.008

*Adjusted R-square 0.73*.

′Number of different medications′ × 1 + ′Number of prescribers′ × 3 + ′Fall incident′ × 7 + ′Does not collect own medication′ × 4. This algorithm has a minimum score of 8, since eligible patients have at least five medications and one prescriber. The maximum algorithm score is dependent on the number of medications and prescribers a patient can have.

### Pilot Study

Of the 400 invited patients, 166 accepted to participate and completed the questionnaire with information needed for the algorithm. Forty patients were randomly selected for a medication review, two of whom dropped out due to illness. Of the remaining patients, 19 were from the lowest and 19 were from the highest tertile of algorithm scores. General patient characteristics of those two groups, such as age and sex, were similar (Table 3).The pharmacists decided to conduct advanced medication reviews for all 38 patients.

**Table 3:** Patient characteristics of the 38 patients who received a medication review.

**Variable**	**Low complexity**	**High complexity**
Total number *(N)*	19	19
Mean age (years)	74.5	76.7
Mean weight (kilogram)	86.4	82.5
Male *(N)*	11	12
Sachet packed medication *(N)*	1	4
At least one unplanned hospitalization in the last 12 months *(N)*	1	6
Mean QOF standard morbidity score (Carey et al., 2013)	0.4	1.1
Mean QOF extended morbidity score (excluding renal disease) (Carey et al., 2013)	0.4	1.2
Mean number of medications	7.5	11.1
Mean number of prescribers	1.5	2.9
Does not collect own medication *(N)*	2	8
At least one fall incident in the last 12 months *(N)*	0	6
Mean score algorithm	12.1	23.9

*QOF, Quality and Outcomes Framework; N, number*.

#### Agreement

Kappa values for agreement on complexity ratings ranged from 0.13 to 0.55 (Table 4). For the pharmacists' ratings, the highest agreement (Kappa of 0.42) was seen for the cut-off point of ≥7 (83.3% of the patients rated with a high complexity of 7 or more by the experts were equally scored by the algorithm). For the experts' ratings, the highest agreement (Kappa of 0.55) was seen for a lower cut-off point of ≥5 (74.3% of the patients rated with a complexity of 5 or more by the pharmacists were equally scored by the algorithm). Agreement on the need for a review between the algorithm scores and the community pharmacists' or the experts' ratings ranged from 0.052 to 0.32 (Table 4). The highest observed Kappa was seen for cut-off point ≥8 for the pharmacists and ≥6 for the experts.

**Table 4:** Agreement of the algorithm score with the community pharmacists' and experts' ratings on complexity and on need for medication review at different cut-off points.

	**Community pharmacists**		**Experts**	
**9-point Likert scale^†^**	**Agreement (%)**	**Kappa (κ)**	**Agreement (%)**	**Kappa (κ)**
**COMPLEXITY RATING**				
≥4	63.1	0.26	73.7	0.47
≥5	68.4	0.37	77.6	0.55
≥6	68.4	0.37	73.7	0.47
≥7	71.1	0.42	61.8	0.24
≥8	57.9	0.16	56.6	0.13
**NEED FOR MEDICATION REVIEW RATING**				
≥4	52.6	0.052	59.2	0.18
≥5	57.9	0.16	61.8	0.24
≥6	63.2	0.26	64.5	0.29
≥7	60.5	0.21	61.8	0.24
≥8	65.8	0.32	55.3	0.11

† *Kappa statistics were calculated for different cut-off points of the 9-point Likert scale, e.g., for the complexity rating at a cut-off at ≥7 all patients rated 7 or higher were categorized in the high complexity group and below 7 in the low complexity group; GP, general practitioner*.

#### Pharmacists' Views on the Algorithm

From the interviews, the following five main themes were identified: (1) views about the criteria included in the algorithm, (2) criteria the pharmacists believed that could be relevant for selection but were not included in the algorithm, (3) views about the criteria as proposed by the Healthcare Inspectorate, (4) feasibility of collecting the data needed to apply the algorithm, (5) reliability of the data needed to apply the algorithm (Table 5).

**Table 5:** Themes with quotes from semi-structured interviews with the pharmacists (translated from Dutch).

**Pharmacist number**	**Quote number**	**Theme with quotes**
**VIEWS ABOUT THE CRITERIA INCLUDED IN THE ALGORITHM**		
Ph1	Q1	Fall incidents are important of course. I thought at first there were a lot of things [criteria] missing, but when I thought about it later it made sense.
Ph2	Q2	That can change over time [collects his/her own medication], but yes, I think that these are good criteria.
Ph3	Q3	Yes, they [criteria] seem good to me, especially fall incidents are something you have to be aware of in older people. (…). I think the most important criteria are in there.
Ph4	Q4	Those fall criteria, I have had such people here. Did you fall? How often in the past year? Yes, I tripped once because I did not see the curb or something. I find that difficult, what to do with that.
Ph4	Q5	Yes, number of medication; that is, of course, that makes it complex.
**POSSIBLY RELEVANT CRITERIA NOT INCLUDED IN THE ALGORITHM**		
Ph2	Q6	What you see in medication reviews is that people are often already in disease programs (…) if people are not in such programs, then you have somebody that you think, there is a lot to do.
**VIEWS ABOUT CRITERIA AS PROPOSED BY THE HEALTHCARE INSPECTORATE**		
Ph3	Q7	I think the criteria of the Inspectorate are okay but on the other hand I rather have somebody who is young with a lot of medication. Then I can do a lot, so that in the future they can use less (…) the older people may have a lower kidney function but I find the younger are really more important.
**FEASIBILITY OF COLLECTING THE DATA NEEDED FOR THE ALGORITHM**		
Ph1	Q8	If you can easily make a selection and list for sending letters, then I think it is feasible (…) The way it was done now worked fine.
Ph2	Q9	Yes everything is possible but is it worth the effort? (…) Especially fall incidents, I think that would take the most effort. That would require the most change.
Ph3	Q10	You would have to approach each patient to ask whether they have fallen recently and I think that would not be something we would do soon. We would rather look for easier things (…) what we have on our computer.
Ph3	Q11	I think if you, for instance, would only exclude the fall incidents from your algorithm you would be able to implement it a lot faster.
Ph4	Q12	We cannot document that. That is just not doable. (…) When patients have to wait for 3 min they get impatient. (…) So I think that is not feasible.
**RELIABILITY OF THE DATA NEEDED FOR THE ALGORITHM**		
Ph2	Q13	I notice that people find such a question [about fall incidents in last 12 months] difficult to answer.
Ph4	Q14	For a lot of criteria, I wonder whether you get the correct information. Fall incidents, I wonder, number of prescribers, I wonder. Number of medication, that is fairly evident.

Three pharmacists were supportive of including “the number of medications” (Table 5, Q5) and two were supportive of “not collecting their own medication” (Q2) as criteria for medication reviews. One pharmacist saw “the number of prescribers” as a relevant criterion. Using “fall incidents” as criterion elicited mixed reactions with two pharmacists seeing this as important (Q1, Q3) while two others saw “fall incidents” as less relevant in clinical practice (Q4). Three pharmacists suggested “living in a care home,” “diminished cognition,” “patients not included in a healthcare program” (e.g., diabetes or cardiovascular risk management) (Q6) and “patients avoiding healthcare” as potential relevant criteria. Two pharmacists were critical of the Healthcare Inspectorate's current criteria. One observed that the high age criterion would exclude younger patients with polypharmacy, who may benefit from a medication review (Q7). Another expressed a strong negative opinion saying that these criteria were ridiculous, since almost no eligible patients can be selected.

Mixed opinions were expressed as to the feasibility of collecting data needed to apply the algorithm. Sending questionnaires to all eligible patients was considered feasible by two pharmacists (Q8) but judged as not worth the effort by one of them (Q9), whilst a third pharmacist stated that the extra step of collecting the required data would take too much time (Q12). A fourth pharmacist preferred the use of criteria easily obtained from the pharmacy information system (Q10). Collecting the information on the recent fall incidents was considered most difficult and time consuming. Three pharmacists mentioned that asking patients could also lead to unreliable data (Q13, Q14).

#### Medication Review Evaluation

The total time involved for all the steps of the review was on average 76.9 min for patients with a high complexity score and 66.1 min for patients with a low complexity score (Table 6). The 11 min difference was not statistically significant (*P* = 0.134).

**Table 6:** Time the pharmacists needed to perform the medication reviews.

**Step in review**	**Low complexity**		**High complexity**		***P*-value**
	**Time (min)**	***SD***	**Time (min)**	***SD***	
Preparation	11.2	5.5	12.2	6.6	0.616
Anamnesis	29.5	11.5	33.7	12.0	0.278
Analysis	14.5	6.9	18.9	7.2	0.057
Consultation with GP	4.7	3.9	6.6	4.4	–
Feedback to patient	6.3	5.7	5.5	4.4	–
Total time	66.1	20.8	76.9	22.4	0.134

*Min, minutes; SD, standard deviation; GP, general practitioner*.

No difference was found in the number of interventions resulting from the medication review. On average, 2.1 interventions (SD 1.2) were proposed for patients with high scores and 2.3 interventions (SD 1.6) for patients with low scores (*P* = 0.658). The number of implemented interventions did not differ between patients with high and low complexity scores (1.6 +/− 1.3 vs. 1.6 +/− 1.2; *P* = 0.51). There was also no difference in the type of proposed interventions (Table 7).

**Table 7:** Number of proposed interventions in the low complexity and the high complexity group.

**Type of intervention**	**Low complexity**	**High complexity**
De-intensification of medication	18	16
Intensification of medication	9	8
Requesting medical test results or therapeutic drug monitoring	7	5
Intervention not otherwise specified	4	3
Substitution of medication	3	2
Watchful waiting	0	3
Medication administration instructions	2	1
Adherence counseling	0	2
Improving usability of medication	1	0

## Discussion

We developed an algorithm to select older people with polypharmacy for different types of medication review based on their case complexity. The algorithm used the following criteria to calculate a score: number of different medications, number of prescribers, collecting own medication, and having had a severe fall in the last 12 months. It explained 73% of the variation in complexity as rated by the experts. The agreement between the participating pharmacists' ratings and the algorithm scores on complexity was slight to moderate. Pharmacists in the pilot study had mixed opinions about the feasibility of applying the algorithm in practice. The time needed to collect the information for the criteria in the algorithm appeared to be a major barrier. There was no significant difference in time needed for a medication review between patients with high and low complexity scores.

The number of prescribed medication, as included in our algorithm, is often used as a selection criterion for medication reviews or other medication risk management interventions (Huiskes et al., 2017). In the backward stepwise regression procedure, we included other more sophisticated medication scores but a simple count of the number of medications was found to be the best predictor. This is in line with research showing that the Medication Regimen Complexity Index (MRCI) and the number of prescribed medications are equally predictive for hospitalization (Wimmer et al., 2016). A clear benefit of using a simple medication count compared to more complex scores is that it can be calculated relatively easily from the pharmacy information systems. Whether or not a patient collects his or her own medication is a new and possibly meaningful criterion for selecting patients. This criterion can be seen as a proxy for frailty and vulnerability of the patient. We found that not collecting his/her own medication was correlated with diminished mobility and a smaller social network in the patients on which the algorithm was based (data not shown). Furthermore, patients who do not collect their own medication have less contact with the pharmacy personnel. Reduced opportunities for pharmacy personnel to communicate with the patient complicate patient counseling and detecting medication problems. Fall risk has been proposed before as criterion for conducting medication reviews (Nederlands Huisartsen Genootschap, 2012). Severe falls in the older population are recognized as an important cause of trauma and hospitalizations (Gelbard et al., 2014). In part, these events are medication-related (de Jong et al., 2013). However, evidence is still limited that the withdrawal of high risk fall medication as a singular intervention reduces the number of falls (Gillespie et al., 2012; Boye et al., 2017). The number of prescribers has not yet been used as criterion for medication reviews. It has been shown, however, that patients with a larger number of prescribers have a higher risk of inappropriate prescriptions (Hajjar et al., 2005). Being under the care of multiple physicians possibly indicates higher clinical complexity. Several criteria such as age or kidney function, that are commonly used for selecting patients for medication review (Huiskes et al., 2017), were not part of our selection algorithm. The algorithm was developed for patients of 65 years or older. Differentiating further on age within this group did not contribute to predicting case complexity. This is in line with research showing that in older populations the number of potentially inappropriate medications is not associated with age but rather with frailty (Wawruch et al., 2008; Ruggiero et al., 2010; Poudel et al., 2014). Impaired kidney function has often been associated with an increased risk of medication-related hospitalizations and adverse drug reactions (Leendertse et al., 2008; Onder et al., 2010; Sikdar et al., 2012). However, in the Dutch setting this might be less relevant as criterion since community pharmacists already monitor medication safety in patients with a reduced kidney function (Koster et al., 2016).

The agreement between the algorithm scores on complexity and the pharmacists' ratings was at best moderate. In particular, several patients with low complexity scores as calculated by the algorithm were rated complex by the pharmacists. Agreement was better regarding patients with high complexity, as indicated by the higher Kappa value when using a cut-off score of 7 or more. This implies that the algorithm can be useful to select the more complex patients for an advanced medication review. Some of the remaining patients classified as simple by the algorithm, however, may also be in need of an advanced medication review based on clinical judgment of the healthcare provider. Nonetheless, there were also patients with low complexity scores that received similar low ratings by the pharmacists. In the current system, they received an advanced medication review but it is expected that such less complex patients can receive a more simple and less time consuming review. Reservations on the feasibility of applying the algorithm in practice were primarily seen for collecting information related to recent fall incidents. In contrast to the other criteria, data on fall incidents cannot be acquired from the pharmacy information system. However, eligible patients could be asked by pharmacy staff about recent falls when dispensing the medication. Implementation of pharmaceutical care services in community pharmacies is often impeded by a lack of time (van Mil et al., 2001; Kaae and Christensen, 2012). Hence, in order to successfully implement a criteria-based algorithm the time investment should be minimized by making the selection process automated where possible. Furthermore, time invested in collecting data for the algorithm may be compensated by performing shorter medication reviews for low complexity patients.

All patients received advanced medication reviews regardless of their complexity, possibly because only advanced reviews are currently recommended and reimbursed in the Netherlands. Consequently, differences in time investment for patients with high or low algorithm scores were small and did not reach statistical significance in our pilot study. Time restrictions are among the most important barriers in performing medication reviews (Niquille et al., 2010). A time difference of more than 10 min for low complexity patients would allow a pharmacist to perform one additional review for every six reviews performed in this group. Less time consuming types of medication review, like a concordance or prescription review, might be sufficient in certain low complexity patients (Blenkinsopp et al., 2012).

### Strengths and Limitations

A strength of our study is that we used the experts' judgments on case complexity to develop the criteria-based algorithm. This allows for the identification of criteria that can be proxies for criteria that may be difficult to acknowledge or measure, and to search for the criteria that provide an independent contribution. For instance, in the univariate analysis an unplanned hospitalization in the last 12 months was a good predictor for the complexity rating of the experts (Appendix II). However, adding unplanned hospitalization to the regression model failed to substantially improve the model. Thus, although an unplanned hospitalization might have been considered a relevant criterion from an expert's point of view, it did not provide additional information in combination with the other criteria used in the model. Secondly, we used a Delphi method with assessments on 9-point Likert scales, which allowed for achieving a group statistical response, and included two expert panels to increase the reliability of the consensus ratings. There were several limitations to this study. Firstly, the algorithm was based on 80 cases of which 21 were modified. Since the modified cases were fairly similar to the original cases, this might have increased the weight that certain characteristics of these cases had on the algorithm. On the other hand, by changing certain characteristics we may have introduced more variety to these characteristics. Although the characteristics that were varied were not significant in the final model some bias in model building cannot be excluded. Secondly, only patients with high and low algorithm scores were invited for a medication review in the pilot study. Patients in the intermediate group did not receive a review, and no conclusions can be made about this group. Thirdly, out of 400 patients invited, 166 were willing to participate, possibly introducing a selection bias. This rate is similar in relation to other Dutch studies in which advanced medication reviews are conducted (Kwint et al., 2012; Willeboordse et al., 2017). Lastly, the algorithm was applied in four community pharmacies in the northern part of the Netherlands. To obtain more generalizable results, a larger study is warranted.

#### Implications for Practice and Research

Although the algorithm performed well in predicting case complexity as rated by the experts, the fair to moderate performance for distinguishing between high and low complexity patients in the pilot study indicates that it should only be used as a screening tool for selecting complex patients for advanced medication reviews. This pilot study is a first step toward a new approach which allows for a more evidence-based differentiation in the type of medication reviews conducted in practice. Given the fact that our algorithm was developed using expert consensus on a set of partly modified cases, it needs to be validated in a larger set of patients. This process may lead to adapting the algorithm to improve its performance. Furthermore, optimal cut-off levels for the degree of complexity in relation to the type of medication review need to be confirmed. Finally, studies should be conducted to assess the effects of this new approach on clinical outcomes.

In conclusion, we developed an algorithm with four criteria that differentiates between high and low complexity patients and allows allocation to simple or advanced medication reviews accordingly. In practice, most feasibility problems were seen regarding collection of information about recent falls. Additional steps are needed to confirm the correctness of the four criteria. Furthermore, steps are needed to facilitate data collection for the algorithm, and to evaluate and improve the algorithm before implementing it in practice.

## Ethics Statement

The Medical Ethics Review Board from the University Medical Center Groningen considered the pilot study as falling outside the scope of the Dutch Medical Research involving Human Subjects Act.

## Author Contributions

SC, JS, JH, MV, MT, KT, and PD: research idea and study design. SC, JS, KT, and PD: data acquisition. SC, KT, and PD: analysis and interpretation. KT and PD: supervision or mentorship. All authors contributed substantially to the intellectual content during manuscript drafting or revision. All authors approved the manuscript and this submission.

### Conflict of Interest Statement

The authors declare that the research was conducted in the absence of any commercial or financial relationships that could be construed as a potential conflict of interest.
