# Supplementary material for: Development and Piloting of an Algorithm to Select Older Patients for Different Types of Medication Review
Source: Front Pharmacol. 2019 Mar 19;10:217. doi: 10.3389/fphar.2019.00217 (PMC6433968; doi:10.3389/fphar.2019.00217)
Supplement: Supplementary file 1 [file Table_1.pdf]

Appendix i. Translated Case Report Form

PATIENT DATA

Age: ☐ year

Weight: ☐ kg

Sex :
- Male ☐
- Female ☐

Living situation:
- Single ☐
- Living together ☐
- Other ………………

Dutch speaking:
- Yes ☐
- No ☐

Receives sachet packed medication:
- Yes ☐
- No ☐

Patient is illiterate or low literate :
- Yes ☐
- No ☐

What is the highest level of education?

- No education / elementary education ☐
- Pre-vocational education ☐
- Vocational training ☐
- Selective secondary education ☐
- University of applied science/university ☐

How may fall incidents did the patient have in the last 12 months?

Not ☐

1 time ☐

2-3 times ☐

≥4 times ☐

How many unplanned hospitalizations did the patient have in the last 12 months?

Not ☐

1 time ☐

2-3 times ☐

≥4 times ☐

How many prescribers does the patient have?

1 (only the general practitioner (GP)) ☐

2 (GP + 1 specialist) ☐

3 (GP + 2 specialists) ☐

4 or more (GP + 3 or more specialists) ☐

Indicate to what extent the following aspects are applicable for this patient:

Eyesight impairment:

Not impaired				Seriously impaired
1	2	3	4	5
☐	☐	☐	☐	☐

Hearing impairment:

Not impaired				Seriously impaired
1	2	3	4	5
☐	☐	☐	☐	☐

Dimished cognition:

Not impaired				Seriously impaired
1	2	3	4	5
☐	☐	☐	☐	☐

Diminished mobility:

Not impaired				Seriously impaired
1	2	3	4	5
☐	☐	☐	☐	☐

Social network:

Adequate social network				No social network
1	2	3	4	5
☐	☐	☐	☐	☐

SIGNALS FROM THE PATIENT

Does the patient have problems with medication adherence?

No problems ☐

Frequently forgets medication ☐

Has difficulties to understand the instructions ☐

Deviates from instructions without consultation ☐

Other, namely:**…………………..**

The patient has indicated that he/she suffers from side effects:

Yes *Complete with information about type and seriousness*

No ☐

The patient has indicated that he/she has problems with the intake of medication (such as swallowing problems)?

Yes, namely: *Complete with type of medication taking problem*
No ☐

The patient has indicated that he/she thinks that the medication is not working:

Yes ☐
No ☐

The patient has indicated that he/she worries about the amount of medication that is prescribed:

Yes ☐
No ☐

The patient collects the medication him/herself at the pharmacy:

Yes ☐
No ☐

The patient has difficulties to communicate about his/her situation:

Yes ☐
No ☐

The patient seeks timely contact with the healthcare professionals when it is needed:

Yes ☐
No ☐

Are there any other relevant background characteristics known

……………..

MOST RECENT ASSESSMENTS

Type of assessment	Outcome value
**Blood pressure**	/ mmHg
**LDL-cholesterol**	mmol/l
**HDL-cholesterol**	mmol/l
**Total cholesterol**	mmol/l
**Total cholesterol/HDL-c**	
**eGFR**	ml/min/1,73m^2
**Sodium**	mmol/L
**Potassium**	mmol/L
**TSH**	mU/l
**Hb1Ac**	mmol/mol
**ALAT**	U/I
**ASAT**	U/L
**Other assessment**	
**Other assessment**	
**Other assessment**	
