# Supplementary material for: Development and Piloting of an Algorithm to Select Older Patients for Different Types of Medication Review
Source: Front Pharmacol. 2019 Mar 19;10:217. doi: 10.3389/fphar.2019.00217 (PMC6433968; doi:10.3389/fphar.2019.00217)
Supplement: Supplementary file 2 [file Table_2.pdf]

Appendix ii. Predictors used in univariate testing for the development of the algorithm, grouped by category, and reasons of exclusion for testing in the multivariate models

Predictor	Description	Univariate analysis	P-value (algorithm score)	Reason of exclusion
**Medication distribution**				
Collects his/her own medication†	Medication is collected by the patient him/herself at the pharmacy.	T-test	0.0020	-
Sachet packed medication‡	Receives sachet packed medication	T-test	0.0045	-
**Mobility**				
Diminished mobility†	Has no to seriously impaired mobility	T-test	<0.0001	-
Diminished mobility (categorised)	Diminished mobility in 5 categories from not to seriously impaired mobility	Oneway anova	<0.0001	No difference in complexity between different categories
**Blood pressure**				
Systolic blood pressure†	Systolic blood pressure	Linear regression	0.0053	-
Low systolic blood pressure‡	Systolic blood pressure below 120 mmHg when under 80 years old and below 140 mmHg when 80 years or older	T-test	0.0107	-
Diastolic blood pressure	Diastolic blood pressure	Linear regression	0.2881	P-value >0.25
High systolic blood pressure	Systolic blood pressure over 140 mmHg when <80 years or Systolic blood pressure over 160 mmHg when ≥80 years	T-test	0.1077	Patients with high blood pressure had a lower algorithm score

High or low systolic blood pressure	Either low or high systolic blood pressure	T-test	0.3005	P-value >0.25
High or low blood pressure + at least 3 antihypertensive medications	Has high or low blood systolic pressure in combination with ≥3 antihypertensive medications	T-test	0.7394	P-value >0.25
Low systolic blood pressure + at least 3 antihypertensive medications	Low systolic blood pressure in combination with ≥3 antihypertensive medications	T-test	0.2841	P-value >0.25
**Medication**				
Number of medications†	A count of all medications with a distinct ATC-code	Linear regression	<0.0001	-
Number of antihypertensive medications	A count of antihypertensive medications	Linear regression	0.3123	P-value >0.25
HARM medication (Leendertse et al., 2008)‡	A count of medications responsible for many medication related hospitalisations according to the HARM study, excluding antibiotics	Linear regression	0.0816	-
HARM+ narrow therapeutic index + high risk medication (Barnett et al., 2011; Blix et al., 2010; Leendertse et al., 2008)	A count of medications responsible for many medication related hospitalisations according to the HARM study (excluding antibiotics) plus other medications considered	Linear regression	0.0062	-

Saedder et al., 2014)‡	to be high risk medication			
ACB anticholinergic score (Campbell et al., 2009)‡	A score of the anticholinergic load of the medications according to the Anticholinergic Cognitive Burden Scale	Linear regression	0.0228	-
Number of medications requiring adjustment according to eGFR	A count of the medications that need to be adjusted in the presence of low eGFR	Linear regression	0.9853	P-value >0.25
MRCI (George et al., 2004)‡	Adjusted Medication regimen complexity index (MRCI)	Linear regression	<0.0001	-
Number of units a day‡	A count of all units (pills, inhalations, injections, etc.) to be used per day	Linear regression	<0.0001	-
**Kidney function**				
eGFR†	Estimated Glomerular Filtration Rate	Linear regression	0.2368	-
eGFR categorised	eGFR in 3 categories: <30 , 30-59 and >59 ml/min/1,73m^2	Oneway anova	0.2729	P-value >0.25
Low eGFR in combination with a relevant medication‡	eGFR <60 ml/min/1,73m^2 in combination with at least 1 medication that needs to be adjusted for eGFR	T-test	0.2448	-
**Hospitalisations**				
Hospitalisation in	Has had at least 1	T-test	0.0047	-

the last 12 months†	unplanned hospitalisation is the last 12 months			
Number of unplanned hospitalisations in last 12 months	Unplanned hospitalisations in 4 categories: none; 1; 2 or 3 ; 4 or more	Oneway anova	0.0253	No difference in complexity between 1 or more hospitalisations
**Morbidity scores**				
QOF (Carey et al., 2013)‡	Quality and Outcomes Framework score	Linear regression	<0.0001	-
QOF extended, excluding renal disease (Carey et al., 2013)‡	Quality and Outcomes Framework extended score	Linear regression	<0.0001	-
QOF extended excluding renal disease (Carey et al., 2013)†	Quality and Outcomes Framework extended score, excluding renal disease	Linear regression	<0.0001	-
**Cholesterol**				
Total cholesterol	Total cholesterol level	Linear regression	0.0511	Patients with a high cholesterol had a lower algorithm score
Total cholesterol/HDL	Total cholesterol divided by High-density-lipoprotein (HDL) level	Linear regression	0.9805	P-value >0.25
**Fall incidents**				
Fall incidents†	Has had at least 1 severe fall incident is the last 12 months	T-test	0.0006	-
Fall incidents categorised	Number of fall incidents in 4 categories: no falls; 1; 2 or 3 ; 4 or more	Oneway anova	0.0018	No difference between 1 or more fall incidents
**Age**				

Age†	Age	Linear regression	0.0483	-
Age categorised	Age in 5 year classes	Oneway anova	0.0409	Differences between groups cannot be explained by older age
**Other individual characteristics (not part of a category)**				
Number of prescribers†	A count of all active prescribers including the GP	Linear regression	<0.0001	-
Diminished eyesight	Diminished eyesight in 5 categories from not to seriously diminished eyesight	Oneway anova	0.1190	Differences between groups cannot be explained by the severity of the eyesight impairment
Diminished social network	Diminished social network in 5 categories from no to adequate social network	Oneway anova	0.0353	Differences between groups cannot be explained by more severely diminished social network
Adherence issue†	Patient has some form of adherence problems	T-test	0.1488	-
Believes that (some of the) medication does not work†	Patient believes that (some of) his/her medication does not work	T-test	0.1683	-
Worries about the amount of medication†	Patient worries about the amount of medication he/she is taking	T-test	0.1974	-
Difficulty in communicating†	Patient has difficulties in communicating (with healthcare professionals)	T-test	0.0818	-
Education	Level of education	Oneway	0.3089	P-value >0.25

	divided in 5 categories	anova		
Deviating potassium or sodium levels	Potassium or sodium level is outside the reference value; potassium [3.5-5.0 mmol/L]; sodium [135-145 mmol/L]	T-test	0.8062	P-value >0.25
Side effect(s)	Reported at least 1 side effect	T-test	0.4841	P-value >0.25
Intake problem(s)	At least 1 issue for medication intake, e.g. swallowing problem, inhalation problem	T-test	0.6834	P-value >0.25
Diminished hearing	Diminished hearing in 5 categories from not to seriously impaired hearing	Oneway anova	0.9416	P-value >0.25
Diminished cognition	Diminished cognition in 5 categories from not to seriously impaired cognition	Oneway anova	0.6479	P-value >0.25
Sex	Male or female	T-test	0.6706	P-value >0.25
Contacts healthcare professionals in time	Patient contacts healthcare professionals in time when needed	T-test	0.3407	P-value >0.25

† Included in the first backward stepwise linear regression procedure,‡ Alternative predictor tested in the model

References

Barnett, K., McCowan, C., Evans, J. M. M., Gillespie, N. D., Davey, P. G., and Fahey, T. (2011). Prevalence and outcomes of use of potentially inappropriate medicines in older people: cohort study stratified by residence in nursing home or in the community. BMJ Qual. Saf. 20, 275–281. doi: 10.1136/bmjqs.2009.039818

Blix, H. S., Viktil, K. K., Moger, T. A., and Reikvam, A. (2010). Drugs with narrow Q11 therapeutic index as indicators in the risk management of hospitalised patients. Pharm. Pract. 8, 50–55. doi: 10.4321/S1886-36552010000100006

Campbell, N., Boustani, M., Limbil, T., Ott, C., Fox, C., Maidment, I., et al. (2009). The cognitive impact of anticholinergics: a clinical review. Clin. Interv. Aging 4:225–233. doi: 10.2147/CIA.S5358

Carey, I. M., Shah, S. M., Harris, T., DeWilde, S., and Cook, D. G. (2013). A new simple primary care morbidity score predicted mortality and better explains between practice variations than the Charlson index. J. Clin. Epidemiol. 66, 436–444. doi: 10.1016/j.jclinepi.2012.10.012

George, J., Phun, Y., Bailey, M. J., Kong, D. C. M., and Stewart, K. (2004). Development and validation of the medication regimen complexity Index. Ann. Pharmacother. 38, 1369–1376. doi: 10.1345/aph.1D479

Leendertse, A. J., Egberts, A. C., Stoker, L. J., and van den Bemt, P. M. (2008). HARM study group frequency of and risk factors for preventable medication related hospital admissions in the Netherlands. Arch. Intern. Med. 168,1890–1896. doi: 10.1001/archinternmed.2008.3

Saedder, E. A., Brock, B., Nielsen, L. P., Bonnerup, D. K., and Lisby, M. (2014). Identifying high-risk medication: a systematic literature review. Eur. J. Clin. Pharmacol. 70, 637–645. doi: 10.1007/s00228-014-1668-z
