# Supplementary material for: Development and Piloting of an Algorithm to Select Older Patients for Different Types of Medication Review
Source: Front Pharmacol. 2019 Mar 19;10:217. doi: 10.3389/fphar.2019.00217 (PMC6433968; doi:10.3389/fphar.2019.00217)
Supplement: Supplementary file 3 [file Table_3.pdf]

Appendix iii. Translated questionnaire used in the pilot study to collect patient information needed for the algorithm

Questionnaire

Dear Mr./Mrs. ……………………………………..

We would like to ask you some questions for the medication review study. If you are not completely sure about an answer, try to answer the question as best as you can. At the end of the questionnaire there is space for remarks.

Question 1.
Do you collect your own medication at the pharmacy?

Yes ☐
No ☐

Question 2.
Do you use medication which is prescribed by a different physician than your general practitioner? For instance, a pulmonologist or a cardiologist from the hospital or a psychiatrist.

Yes ☐
No ☐

In case of a yes:
How many different physicians besides your general practitioner prescribe your medication?

……

Question 3.
In the last 12 months, have you had a fall so severe that you needed help from other people? For instance, you needed to go to the general practitioner or the emergency room because of this fall. Or you needed extra help in or around the house because of this fall.

Yes ☐
No ☐

Any questions or remarks can be written down below.
