# Supplementary material for: Development and Piloting of an Algorithm to Select Older Patients for Different Types of Medication Review
Source: Front Pharmacol. 2019 Mar 19;10:217. doi: 10.3389/fphar.2019.00217 (PMC6433968; doi:10.3389/fphar.2019.00217)
Supplement: Supplementary file 4 [file Table_4.pdf]

Appendix iv. Topic list for the interviews performed with the community pharmacist in the pilot study

Setting and collaboration:	Size of the pharmacy<br>Collaboration with the GP<br>Pharmacotherapy audit meetings
Medication reviews:	Selection of patients<br>Procedure<br>Personnel involved
Usefulness of the algorithm:	Opinion about the four criteria included in the algorithm<br>Differences between reviews performed in both groups<br>Differences in interventions<br>Differences in patients<br>Patients potentially missed by the algorithm
Feasibility:	Feasibility of collecting information for the algorithm<br>Opportunities for automatization<br>Barriers to perform review for the patients selected by the algorithm
